# Cyclic Peptide Inhibitors of the β-Sliding Clamp in *Staphylococcus aureus*

**DOI:** 10.1371/journal.pone.0072273

**Published:** 2013-09-04

**Authors:** Susanne Kjelstrup, Paula Melo Paulon Hansen, Line E. Thomsen, Paul Robert Hansen, Anders Løbner-Olesen

**Affiliations:** 1 Department of Biology, University of Copenhagen, Copenhagen, Denmark; 2 Department of Veterinary Disease Biology, University of Copenhagen, Copenhagen, Denmark; 3 Department of Drug Design and Pharmacology, University of Copenhagen, Copenhagen, Denmark; University of Manchester, United Kingdom

## Abstract

Interaction between pairs of *Staphylococcus aureus* replication proteins was detected in an *Escherichia coli* based two-hybrid analysis. A reverse two-hybrid system was constructed for selection of compounds that hindered interaction between interacting protein pairs. A number of cyclic peptides, from a library generated by the split intein-mediated circular ligation of peptides and proteins technology, were found to interfere with dimerization of the β-sliding clamp of the replisome. Two 8-mer peptides were analyzed in more detail. Both inhibited DNA replication, led to SOS induction, altered cell morphology and cell death. The peptides were active when added to bacterial cultures indicating that they could traverse the bacterial membrane to find their intracellular target. Peptide specificity was confirmed by overproduction of the putative target (DnaN) which resulted in resistance. The minimum inhibitory concentration was ∼50 μg/ml for *S. aureus* cells. These compounds may serve as lead candidates for future development into novel classes of antibiotics as well as provide information on the function of the *S. aureus* replication process.

## Introduction

In recent years, many bacterial pathogens have become resistant or insensitive to most of the currently available antibiotics. As a consequence, infections caused by drug-resistant bacteria, including the Gram-positive methicillin-resistant *Staphylococcus aureus* (MRSA) and vancomycin-resistant *Enterococci* (VRE) are associated with increased morbidity, mortality and health-care costs. The resistance problem has traditionally been addressed by development of semi-synthetic penicillins and the introduction into clinical use of novel antibiotic classes. This development peaked in the 1960’s, and only two new classes of antibiotics, the oxazolidinones and daptomycin [1], [2], have been marketed within the last 30 years. In order to address the limited treatment options for several bacterial infections it is important that the development of antimicrobials continue and include both new targets for intervention as well as new classes of inhibitors.

Chromosome duplication is an essential process in all living organisms and the multienzyme machinery that replicates bacterial DNA represents one such underexploited target. In bacteria the replication process is carried out by highly conserved proteins, which deviate from their eukaryotic counterparts in structure and sequence (reviewed by [3]). Compounds that target bacterial DNA replication are therefore expected to have a high therapeutic index. Most of our current knowledge on bacterial chromosome replication comes from studies of *E. coli*. The DnaA replication initiator protein is an AAA+ protein that binds either ATP or ADP. DnaA associated with either nucleotide binds a number of high affinity sites in the *E. coli* replication origin, *oriC*, throughout the cell cycle to form the pre-replicative complex [4]. Formation of a DnaA-ATP sub-complex at the binding sites in the left half of *oriC* and flanking the DUE (Duplex Unwinding Element) region is essential for helicase loading, and is stimulated by the formation of a second DnaA sub-complex in the right half of *oriC* [5]. At initiation DnaA-ATP molecules cooperatively bind the left half of the origin to form a right-handed DnaA-ATP helix, where individual DnaA molecules interact through their AAA+ domains [5], [6], with *oriC* DNA wrapped around it. Binding of IHF immediately upstream of the DUE flanking R1 DnaA-box introduces a 160° bend in the DNA reversing the orientation of the DNA helical axis and assist in melting the DUE region. One of the exposed single-stranded DUE regions is fixed by binding the existing DnaA-ATP helix while the other strand is exposed for DnaC assisted DnaB helicase loading by the DnaA molecule bound to the R1 box. Further opening of the duplex allows for loading of the second helicase by one or more N-terminal domains of the DnaA-ATP filament [5]. Although promoted by formation of a DnaA oligomer on *oriC*, the exact mechanism for helicase loading at the origin differ between bacteria (for review see [7]). After helicase loading, a cascade of events leading to replisome assembly and the beginning of the elongation follows [8]. The replisome structure was recently covered in an excellent review [3] and consists of a primosome complex and a PolIII holoenzyme complex, where each PolIII holoenzyme complex can be further divided into three different complexes: PolIII core (αεθ), the sliding clamp (β_2_) and the clamp loader (τ_3_δδ'ψχ). The core polymerase needs the sliding clamp for processivity, which in turn is loaded onto the DNA by the clamp loader.

In the firmicutes including *S. aureus*, the process of elongation is similar to that in *E. coli* with a couple of notable exceptions. The *S. aureus* helicase (called DnaC) is loaded by the DnaI helicase loader assisted by the DnaB and DnaD proteins [9] and two different replicative polymerases are used. The DnaE which is homologous to the *E. coli* PolIIIα only extends RNA primers initially and hands them off to PolC which is responsible for the processive synthesis (reviewed in [10]). A third difference was recently revealed. Primer hand off in *Bacillus subtilis*, can occur after the synthesis of only two nucleotides by the DnaG primase [11] and does not require other replication proteins. This is in contrast to the three-point switch hand off mechanism in *E. coli*. Here the χ polypeptide of the clamp loader interacts with SSB to displace DnaG from the SSB-DnaG complex resulting in release of the primer which is then extended by the processive polymerase [12].

In all bacteria examined so far the ring shaped β-clamp is a homodimer which encircles the DNA and slides along the duplex bringing the polymerase into contact with the DNA to ensure processivity [13]. The β-clamp interacts with many different proteins including DnaE, PolC, δ, PolIV (DinB), PolV (UmuC/D), PolI, MutS, MutL, DNA ligase and Hda. These proteins all contain a conserved β-binding motif (QL^S^/_P_LPL or QL^D^/_S_LF) which binds a hydrophobic pocket located in each DnaN protomer. The β-sliding clamp has been the target for potential new antibiotics and two different approaches have been used to identify compounds that block the peptide-binding pocket of β. First, synthetic peptides containing the beta-binding domain QL^D^/_S_LF were found to inhibit PolC-β2 and δ-β2 interactions [14] and similarly peptides containing β-binding sequence from δ and Hda bound the β-clamp and inhibited DNA synthesis *in vitro* [15]. Subsequently more efficient binders were identified by modification of the β-binding domain [16], [17] and these optimized peptide motifs have served as starting point for small molecule mimics to identify compounds that inhibit the α-β2 interaction at micromolar concentrations [17]. In the second approach, a fluorescence based peptide displacement assay was used to identify small compounds that bind to the peptide-binding pocket of β [18]. One compound, RU7, which inhibited PolII, PolIII and PolIV although to different extents was identified from a collection of 30,600 polar organic compounds. It was suggested that RU7 can be used as a starting point for rational drug design to create stronger inhibitors of replication.

A fairly unexploited class of compounds that has attracted attention as putative antimicrobials is peptides. The extensively studied natural antimicrobial peptides are produced by multicellular organisms and the majority act by insertion and alteration/damage of cytoplasmic membranes via formation of ion channels or transmembrane pores, but other have been associated with intracellular targets such as DNA and RNA synthesis and inhibition of enzymatic activities [19], [20]. This indicates that certain peptides can traverse the bacterial membrane to find their intracellular targets. This suggests that synthetic peptides may be tailored for use as inhibitors of intracellular targets, as proven for synthetic linear peptides targeting holiday junction resolution [21]. A major limitation for the clinical use of antimicrobial peptides is poor proteolytic stability. This may in part be overcome by cyclization, which also confers conformation which may also influence the biological activity of the peptides [22]–[24].

Here we report the identification of small cyclic peptides with the ability to prevent dimerization of the β-clamp and hence DNA replication in *S. aureus*. Peptide circularization *in vivo* was achieved by manipulation of protein splicing (SICLOPPS; split intein-mediated circular ligation of peptides and proteins) which utilizes the DnaE split intein of *Synechocystic* sp. PCC6803 [23], [25]–[28]. This method coupled to reverse bacterial two-hybrid system allowed us to select peptides that were able to decrease protein-protein interactions of selected pairs of replication proteins. Peptides targeting DnaN-DnaN interaction were further characterized with respect to target specificity and activity. A similar approach has earlier been used to identify cyclic peptides that inhibit the *E. coli* ribonucleotide reductase by hampering association between NrdA and NrdB subunits [29].

## Results

Protein-protein interactions in the replicative DNA polymerase and its loaders have been extensively characterized by biochemical and biophysical approaches. In order to demonstrate *in vivo* interactions between *S. aureus* replication proteins in *E. coli* we used the bacterial two hybrid (BTH) system developed by Karimova et al. [30]. This system is based on interaction-mediated reconstruction of adenylate cyclase activity in the adenylate cyclase deficient *E. coli* strain BTH101 (Table 1). In this system the Cya protein of *Bordatella pertussis* is split into two domains (T18 and T25) resulting in loss of activity. If T18 and T25 are fused to interacting polypeptides the two Cya domains will be brought into proximity of each other to create a Cya+ phenotype. This results in cAMP production and consequently in activation of cAMP-CAP regulated promoters (e.g the *lac* promoter).

**Table 1:** Bacterial strains.

Strain/plasmid	Genotype/plasmid properties	Reference
MG1655	Wild-type *E. coli*	[57]
KG22	C600 *lacI^q^ lacZ*Δ*M15*	[58]
DH10B	F^–^ *mcr*A Δ (*mrr*-*hsd*RMS-*mcr*BC) Φ80*lac*ZΔM15 Δ*lac*X74 *rec*A1 *end*A1 *ara*D139 Δ (*ara leu*) 7697 *gal*U *gal*K *rps*L *nup*G λ^–^	[59]
BTH101	F^−^, *cya-99*, *araD139, galE15, galK16, rpsL1 (Str^R^)*, *hsdR2, mcrA1, mcrB1.*	[30]
BTH101Δ*pyrF*	Δ*pyrF*	This work
SC01	BTH101Δ*pyrF*, p*lacZ* ::*pyrF*	This work
RN4220	Restriction-defective derivative of *S*. *aureus* RN450	[60]
8325-4	*S. aureus*	[61]
MTH157	*S. aureus*, *recA*-*lacZ* transcriptional fusion	[62]

We fused *holA*, *holB*, *dnaA*, *dnaB*, *dnaN*, *dnaX* and *polC* of *S. aureus* to the T18 and T25 fragments of Cya from *B. pertussis*. Plasmid pairs were transformed into BTH101 to detect interacting partner proteins. We observed detectable interaction between the β-clamp (encoded by *dnaN*) and the clamp loader (encoded by *dnaX*, *holA* and *holB*) as well as between the components of the clamp loader (Table 2). PolC interacted with the β-clamp and DnaX of the clamp loader. Furthermore, the following interactions were observed: PolC-PolC, DnaN-DnaN, DnaX-DnaX, DnaB-DnaB and DnaA-DnaA. The DnaA-DnaA interaction resulted in very pale blue colonies indicating weak interaction in this assay (Table 2). Growth of *E. coli* cells expressing either of these *S. aureus* replication proteins was not affected. This suggests that none of these proteins interfere negatively with their *E. coli* counterparts. We failed to construct fusions between DnaC and either Cya fragment suggesting that these are toxic to their *E. coli* hosts.

**Table 2:** Protein-protein interactions between the *S. aureus* replicative proteins determined in the BTH system.

T18/T25	HolA (δ)	HolB (δ `)	DnaA	DnaB	DnaN (β)	DnaX (τ)	PolC	Vector	Zip
**HolA (δ)**	−								
**HolB (δ `)**	+	+							
**DnaA**	−	−	+						
**DnaB**	−	−	−	++					
**DnaN (β)**	+	++	−	−	+++				
**DnaX (τ)**	−	++	−	−	++	++			
**PolC**	−	−	−	−	++	++	++		
**Vector**	−	−	−	−	−	−	−	−	
**Zip**	−	−	−	−	−	−	−	−	+++

The relative strength of protein-protein interaction was determined as β-galactosidase level in the BTH assay. (−) white colonies, (+) light blue colonies, (++) blue colonies and (+++) dark blue colonies.

### Selection for compounds that disrupt protein-protein interaction

**Figure 1:**
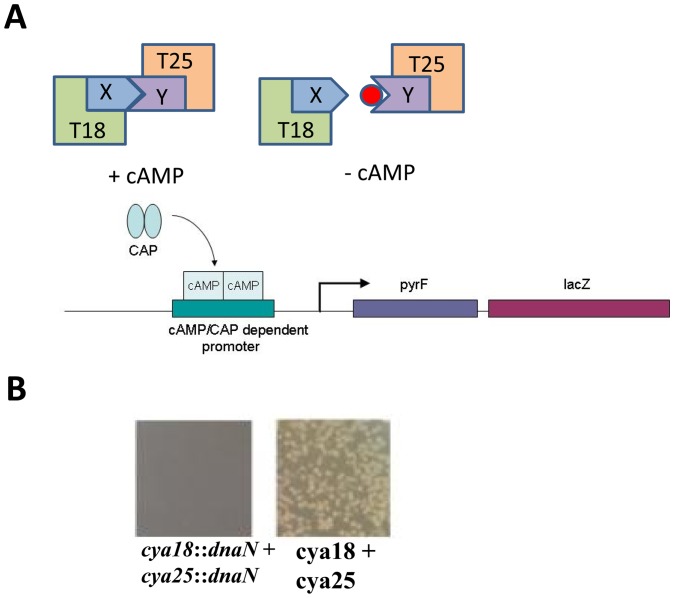
Reverse bacterial two hybrid system. **A**: In the *E. coli* two-hybrid interacting proteins X and Y fused to *B. pertussis* adenylate cyclease T18 and T25 fragments will restore enzyme activity, resulting in cAMP production, and in association with the catabolite activator protein (CAP) activation of cAMP dependent promoters In the reverse two-hybrid system, the *pyrF* gene was placed downstream of the *lac* promoter, hence activation by cAMP results in *pyrF* expression. The *pyrF* gene encodes orotidine-5 ′-phosphate decarboxylase which can convert 5-fluoroorotic acid (5-FOA) to the toxic compound 5-flourouracil whuich results in inviability. If the interaction between X and Y is abolished by a small compound (filled red circle) cAMP will no longer be produced and viability restored. **B**: Fusion of DnaN to both T18 and T25 results in inhibition of growth on 5-FOA plates due to reconstitution of a functional Cya protein. In the absence of fusion partners T18 and T25 will not form a functional andenylate cyclase. Therefore *pyrF* is not expressed and cells remain viable in the presence of 5-FOA.

To directly select for compounds that prevent specific protein-protein interactions we developed a reverse BTH (R-BTH) system based on 5-fluoroorotic acid (5-FOA) selection of PyrF^−^ cells (Fig. 1A). The non-toxic compound 5-FOA is converted to the toxic 5-flurouracil by orotidine-5′-phosphate decarboxylase, the product of the *E. coli pyrF* gene. Bacterial PyrF^+^ cells are therefore not able to grow in rich medium containing 5-FOA, whereas PyrF^−^cells are.

We moved the *pyrF* gene from its original position on the chromosome and placed it in front of *lacZ* in the BTH101 strain, resulting in strain SC01. Interaction between the T18 and T25 fusion proteins results in expression of *pyrF* and consequently inhibition of growth on 5-FOA containing LB plates (Fig. 1B).

We initially tested the R-BTH system with T18 and T25 vectors without fusion partners. This did not result in a PyrF^+^ phenotype and hence growth was observed in the presence of 5-FOA. On the other hand, the same vectors fused to strong interaction partners (ZIP motif) resulted in a PyrF^+^ phenotype and inviability in the presence of 5-FOA (Table 3).

**Table 3:** Protein pairs that can be targeted by the R-BTH system.

	Interaction^a^	Growth on 5-FOA plates^b^
**DnaN-HolA**	+	+
**DnaN-HolB**	++	+
**DnaN-DnaN**	+++	−
**DnaN-DnaX**	++	+
**DnaN-PolC**	++	−
**DnaX-HolB**	++	+
**DnaX-DnaX**	++	−
**DnaX-PolC**	++	−
**DnaA-DnaA**	+	+
**DnaB-DnaB**	++	−
**PolC-PolC**	++	+
**HolB-HolA**	+	+
**HolB-HolB**	+	+
**Vectors**	−	+
**Zip-Zip**	+++	−

a Strength of protein-protein interaction was obtained as in Table 2.

b Was determined by plating on LB plates containing 1.3 μg/ml of 5-FOA. – indicates no growth, whereas + indicates appearance of colonies after 2 days incubation at 30°C.

Interacting pairs of proteins that did not promote growth on 5-FOA plates can be used in a selection for inhibitory compounds.

To determine whether the R-BTH system could be used to select for compounds that prevent interaction between *S. aureus* replication proteins we tested all the interacting protein pairs previously identified (Table 2). Eight sets of interacting proteins (DnaN-HolA, DnaN-HolB, DnaN-DnaX, DnaX-HolB, DnaA-DnaA, PolC-PolC, HolB-HolA and HolB-HolB) did not result in growth inhibition of the R-BTH strain SC01 in the presence of 5-FOA (Table 3). On the other hand, five sets of interacting proteins (PolC-DnaX, PolC-DnaN, DnaN-DnaN, DnaX-DnaX and DnaB-DnaB) all resulted in a PyrF^+^ phenotype and hence inability to grow in the presence of 5-FOA. Thus, we conclude that we can use our R-BTH system to select for compounds that prevent interactions between these five pairs of *S. aureus* replication proteins when expressed in *E. coli*.

### Intracellular production of cyclic peptides

We used the SICLOPPS technology for intracellular synthesis of cyclic peptide libraries [23]. Cyclic peptides were chosen over their linear counterparts to limit degradation by cellular proteases. We initially tested the SICLOPPS system by inserting the coding region for amino acids 1−86 (Domain I) of DnaA from *E.coli* between the C- and N-terminal parts of the split intein.

**Figure 2:**
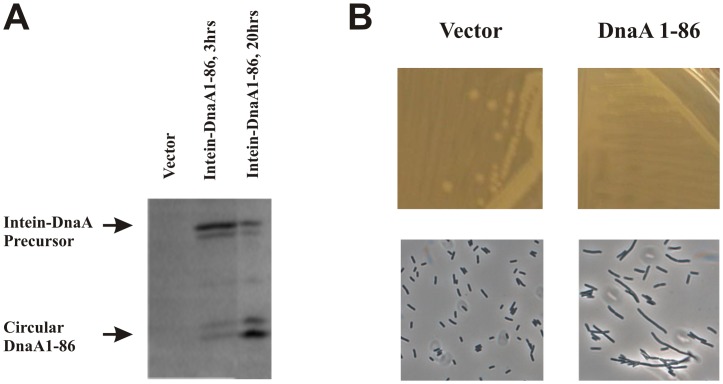
Intracellular production of cyclic DnaA1-86. Expression of IntC::DnaA1−86::IntN was induced by addition of 2 mM IPTG to cells growing exponentially at 30°C or by plating exponential growing cells on plates containing 2 mM IPTG followed by incubation at 30C. **A.** Intein-DnaA precursor and splice products were visualized by Western blot using polyclonal anti-DnaA antibodies. **B.** Growth on LB plates containing 2 mM IPTG (top) and phase-contrast images (bottom) of expressing IntC::IntN (left) or after three hours expression of IntC::DnaA1−86::IntN (right).

Induction of the intein-DnaA1-86 fusion resulted initially (after 3 hrs) in production of two protein bands of approximate size 28 kD, which presumably corresponds to the unspliced fusion protein (Fig. 2A). Two faster migrating protein bands of approximately 10 kD were also observed, albeit in lower amounts. These presumably represent the spliced and cyclic DnaA1−86 fragment. A longer induction time (20 hrs) resulted in an increased ratio of circular DnaA1−86/precursor (Fig. 2A). We do not know the reason for both precursor and splice product appearing as double bands.

Expression of the intein-DnaA1−86 fusion resulted in inhibition of growth and cell filamentation (Fig. 2B). Because all cells contained a mix of precursor and splice product it was not clear which species were responsible for filamentation. We therefore mutated the splice site at the IntC-DnaA1−86 (IntC-HNS-DnaA1−86 to IntC-QYS-DnaA1−86) junction to prevent splicing. Expression of the presumably splice-deficient precursor did not majorly affect cell growth or morphology (not shown), and we can conclude that the growth inhibition observed (Fig. 2B) mainly result from the cyclic DnaA1−86 protein fragment. Although recent biochemical and structural data indicate that domain III and IV of DnaA are responsible for forming DnaA oligomers at *oriC* [6], [31], [32], Domain I was also reported to be involved in oligomerization in addition to its well-recognized role in helicase loading [7], [8], [33], [34], Expression of Domain I may therefore interfere with replication initiation either at the level of open complex formation or helicase loading [33], [35].

### Identification of cyclic peptides that inhibit DnaN-DnaN interaction

Having established the SICLOPPS system for intracellular production of cyclic peptides, we proceeded to construct a peptide library where the produced 21 amino acids (aa) contain a 6 aa randomized sequence. Peptides of 6 aa were previously isolated to inhibit holiday junction resolution [36] and we assumed that this length would be sufficient for our use as well. The library contains 900,000 combinations, of cyclic peptides with the sequence SIIDSAGNNNNNNGASTSESG.

**Figure 3:**
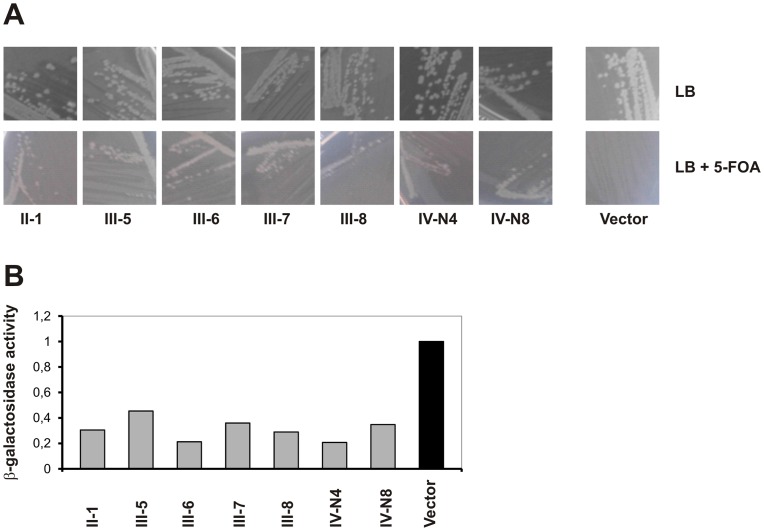
Identification of peptides that interfere with *DnaN*-DnaN interaction. In the reverse two-hybrid strain (SC01) interaction between T18:DnaN and DnaN:T25 resulted in inability to grow in the presence of 5-FOA (A panel; vector). After screening of our SICLOPPS library, growth was restored in 7 clones.A. (B) The cyclic peptides identified in (A) were expressed in the original bacterial two-hybrid strain (BTH101) containing T18:DnaN and DnaN:T25 fusion plasmids and the level of β-galactosidase activity was determined as described in Materials and Methods.

The library was screened for peptides able to disrupt DnaN-DnaN interaction of the *Staphylococcus* replisome by transforming into the R-BTH strain SC01 containing interacting Cya fusion proteins. Cells were plated on plates containing 5-FOA and 1 mM IPTG to induce expression of the cyclic peptides. We readily identified clones where expressed peptides restored cell viability in the presence of 5-FOA (Fig. 3A). To initially assess the activity of the selected peptides we determined their ability to reduce DnaN-DnaN interaction in the original two hybrid system. The β-galactosidase activities measured were reduced to 20−40% of the initial level by all selected peptides, demonstrating that these efficiently reduced dimerization of the *S. aureus* DnaN proteins in *E. coli* (Fig. 3B). The peptide sequences were determined by sequencing of the expression plasmids and are shown in Table 4.

**Table 4:** Peptides identified that target the β-sliding clamp.

Name	Sequence^1^	Purified/synthesized as:	MIC for linear peptide μg/ml (mM)	MIC for cyclic peptide μg/ml (μM)
II-1	WAGSWG	NP	ND	ND
III-5	VFLCGC	**CR**VFLCGC^2^	>683^4^ (0.76)	50^5^ (57)
III-6	SQGLFK	**CR**SQGLFK^2^	>1228^4^ (1.31)	50^5^ (54)
III-7	GHVWVD	**CR**GHVWVD^3^	>673^4^ (0.69)	20^4^ (21)
III-8	STFESL	**CR**STFESL^2^	ND	>100 (>108)
IV-N4	FADCQE	NP	ND	ND
IV-N8	CWLFVL	**CR**CWLFVL^3^	>1124^4^ (>1.08)	>100^4,6^ (>98)

1 Selected as SIIDSAGXXXXXXGASTSESG.

2 Purified using the Impact Twin System (New England Biolabs).

3 Synthesized via FMOC SPPS.

4 Determined for *S. epidermidis*.

5 Determined for *S. aureus*.

6 Although the MIC value above the highest concentration tested (100 μg/ml), growth was severely compromised at concentrations from 25 μg/ml and up.

NP Not produced.

ND Not determined.

### Peptide activities

All of the peptides originally identified as inhibitors of DnaN dimerization came from our 21-mer library (Table 4). Four of these, III-5, III-6, III-7 and IV-N8 were subsequently reduced to 8-mers with the sequence SXXXXXXG while retaining their ability to interfere with DnaN dimerization (not shown). Therefore the activity followed the sequence of the 6 amino acids that were randomized in the libraries. We proceeded to purify peptides III-5, III-6 and III-8 using the pTWIN system (New England Biolabs). Due to the nature of this system, the sequences of purified peptides were CRXXXXXX. Other peptides such as III-7 and IV-N8 were synthesized chemically (Table 4). The proper purity of and structure of peptides was determined by gel electrophoresis and mass spectrometry (not shown).

The antibacterial activities of the purified peptides were determined against *Staphylococcus epidermidis* and *S. aureus*. Cyclic peptides III-5 and III-6 both had MIC values against *S. aureus* of approximately 50 μg/ml whereas they were somewhat more potent against *S. epidermidis* (not shown). Cyclic peptide III-7 was only tested against *S. epidermidis* and had a MIC value of approximately 20 μg/ml (Table 4). Peptide III-8 which also was efficient in reducing DnaN-DnaN interaction when produced intracellularly failed to inhibit *S. aureus* growth at 100 μg/ml, which was the highest concentration tested (Table 4). We can conclude that some but not all of the identified cyclic peptides are able to penetrate the bacterial membrane to find their intracellular target. When we tested the linear counterparts of our isolated peptides they all had MIC values above 560 μg/ml against *S. epidermidis*, indicating little or no activity. As expected none of the peptides isolated had any activity towards *E. coli*, whereas they inhibited growth of *B. subtilis*, another gram positive bacterium (data not shown).

### Overexpression of DnaN in *S. aureus* results in resistance towards peptides III-5 and III-6

**Figure 4:**
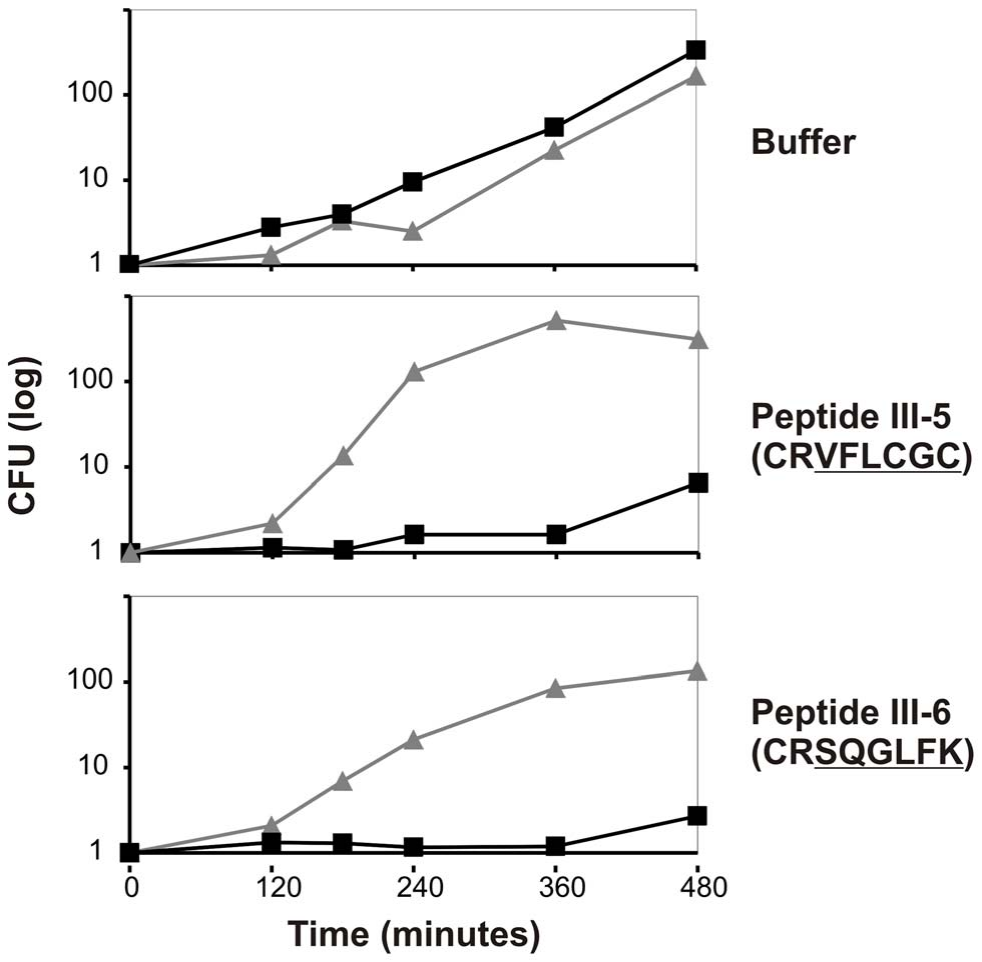
Overproduction of DnaN in *S. aureus* relieves the growth inhibition imposed by peptides III-5 and III-5. *S. aureus* strain 8325-4 containing either the vector plasmid pCN51 (black filled squares) or plasmid pSC141 (grey triangles) was grown exponentially at 37°C in LB medium supplemented with 10 μg/ml erythromycin. CdCl_2_ was added to all cultures to a final concentration of 10 μM to induce overproduction of DnaN from pSC141 three hours prior to peptide addition. At T = 0, 40 μg/ml of peptide III-5 (middle panel) or III-6 (bottom panel) was added to the cultures. The number of colony forming units was determined by plating. The experiment were repeated 4 times with similar results. The figure shows one representative experiment.

To ensure that the antibacterial effects of peptides III-5 and III-6 were the result of direct interaction of these peptides with the β-clamp, we decided to overproduce DnaN in *S. aureus* cells. The *dnaN* gene was cloned under control of the cadmium induclible pCAD promoter of plasmid pCN51 [37]. Cells were grown exponentially at 37°C in LB medium and 3 hours prior to peptide addition expression of *dnaN* was induced by addition of 10 μM CdCl_2_. At time T = 0 peptides III-5 or III-6 were added to a final concentration of 40 μg/ml which is very close to the MIC value for both of these peptides (Fig. 4). Addition of either peptide led to a cessation of bacterial growth of the uninduced cultures for the 8 hours duration of the experiment. On the other hand cultures that had been induced with cadmium and hence overproduced DnaN continued growth in the presence of either peptide (Fig. 4).

We can therefore conclude that overproduction of the putative target for peptides III-5 and III-6 results in resistance towards the peptides, and their antibacterial effect is therefore likely to result from direct interaction with the DnaN protein.

### Peptide III-5 and III-6 inhibit DNA replication

**Figure 5:**
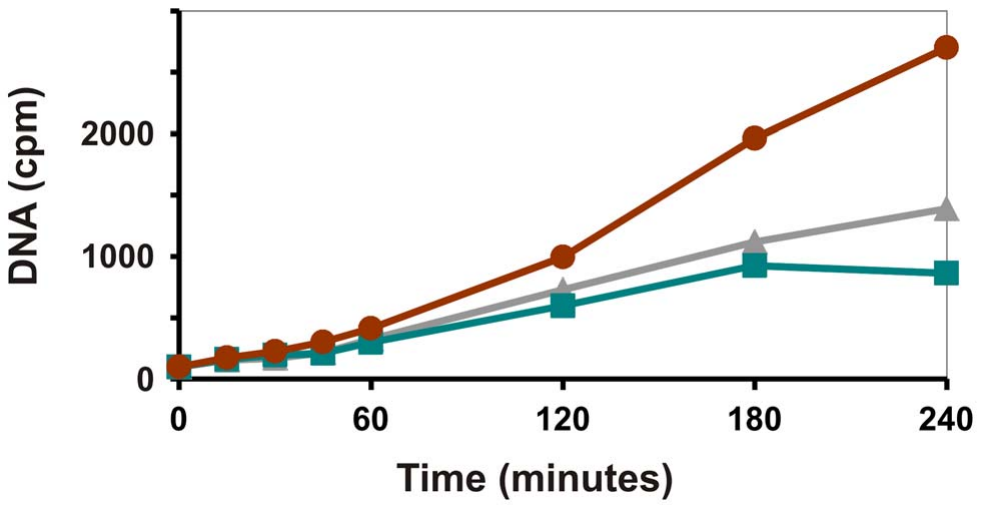
Peptides III-5 and III-6 Inhibit DNA replication. *S. aureus* strain 8325-4 was grown exponentially in LB supplemented with 50 μg/ml uridine and ^3^H-thymidine as described in Materials and Methods. At T = 0 peptide III-5 (grey triangles) or III-6 (green squares) was added to a final concentration of 50 μg/ml. Addition of buffer (red filled circles) served as control. Samples were taken at the indicated time-points and incorporation of ^3^H into DNA was measured by liquid scintillation counting of TCA precipitated material. The experiment were repeated 3 times with similar results. The figure shows one representative experiment.

In order to test the direct effects of peptides III-5 and III-6 on DNA replication in *S. aureus*, strain 8325-4 was grown exponentially at 37°C in LB medium. Incorporation of ^3^H-thymidine into the DNA was determined at various times after addition of 50 μg/ml of peptides III-5 or III-6. Both peptides severely reduced accumulation of DNA (Fig. 5). Protein synthesis, determined as incorporation of ^35^S-methionine, was unaffected by peptide addition for at least 180 minutes (Fig. S1) which is well after the onset of DNA synthesis inhibition. Therefore the DNA replication inhibition is not a consequence of a more general effect on protein synthesis, and we conclude that peptide III-5 and III-6 inhibits replication by interfering with the *dnaN* encoded β-clamp.

**Figure 6:**
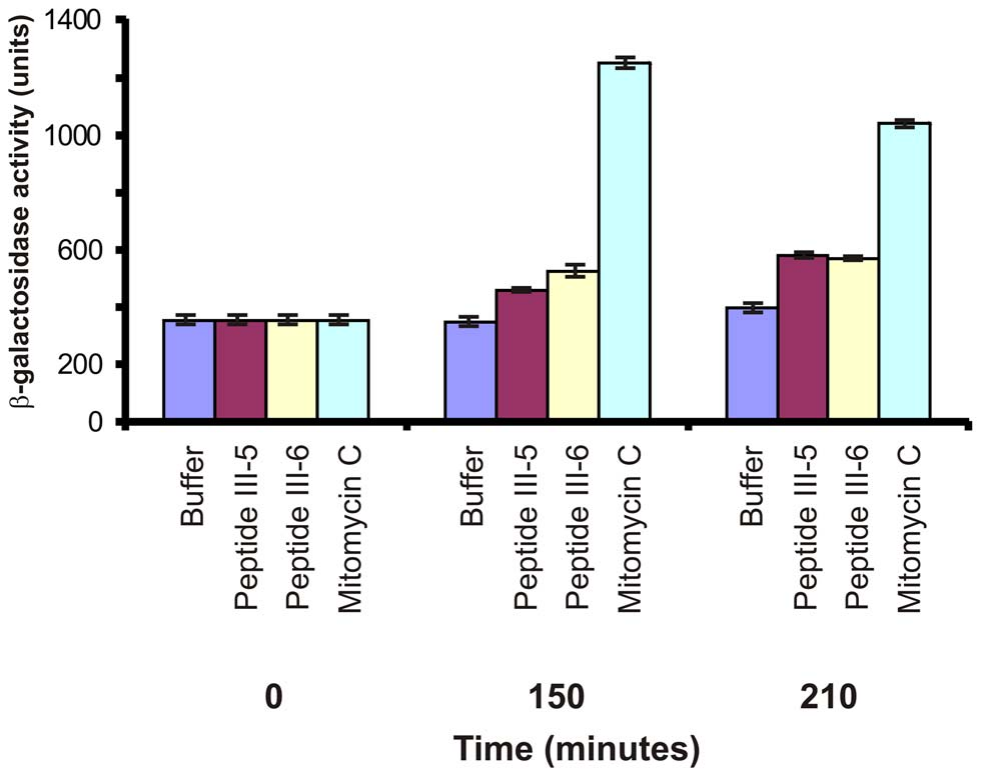
Peptide III-5 and III-6 induces the SOS response in *S. aureus*. *S. aureus* strain MTH157 (*recA*::*lacZ*) was grown exponentially in LB medium at 37°C. At an optical density OD_600_ = 0.1, Mitomycin C or Peptides III-5/III-6 were added to final concentrations of 2 μg/ml or 50 μg/ml, respectively. At the times indicated, samples were taken and the level of β-galactosidase determined (Materials and Methods).

To test whether the cessation of DNA accumulation was accompanied by induction of the SOS response we measured the effects of both peptides on induction of the *recA* gene. Strain MTC157 contains a *recA-lacZ* transcriptional fusion in its chromosome. Addition of either peptide to this strain resulted in a ∼50% increase in β-galactosidase activity after 210 minutes (Fig. 6). The level of induction was less that that observed for the DNA damaging agent mitomycin C (about 3-fold increase after 210 minutes). It is likely that both peptides results in malfunctioning of the replisome, and this provides the signal for SOS induction in these cells.

**Figure 7:**
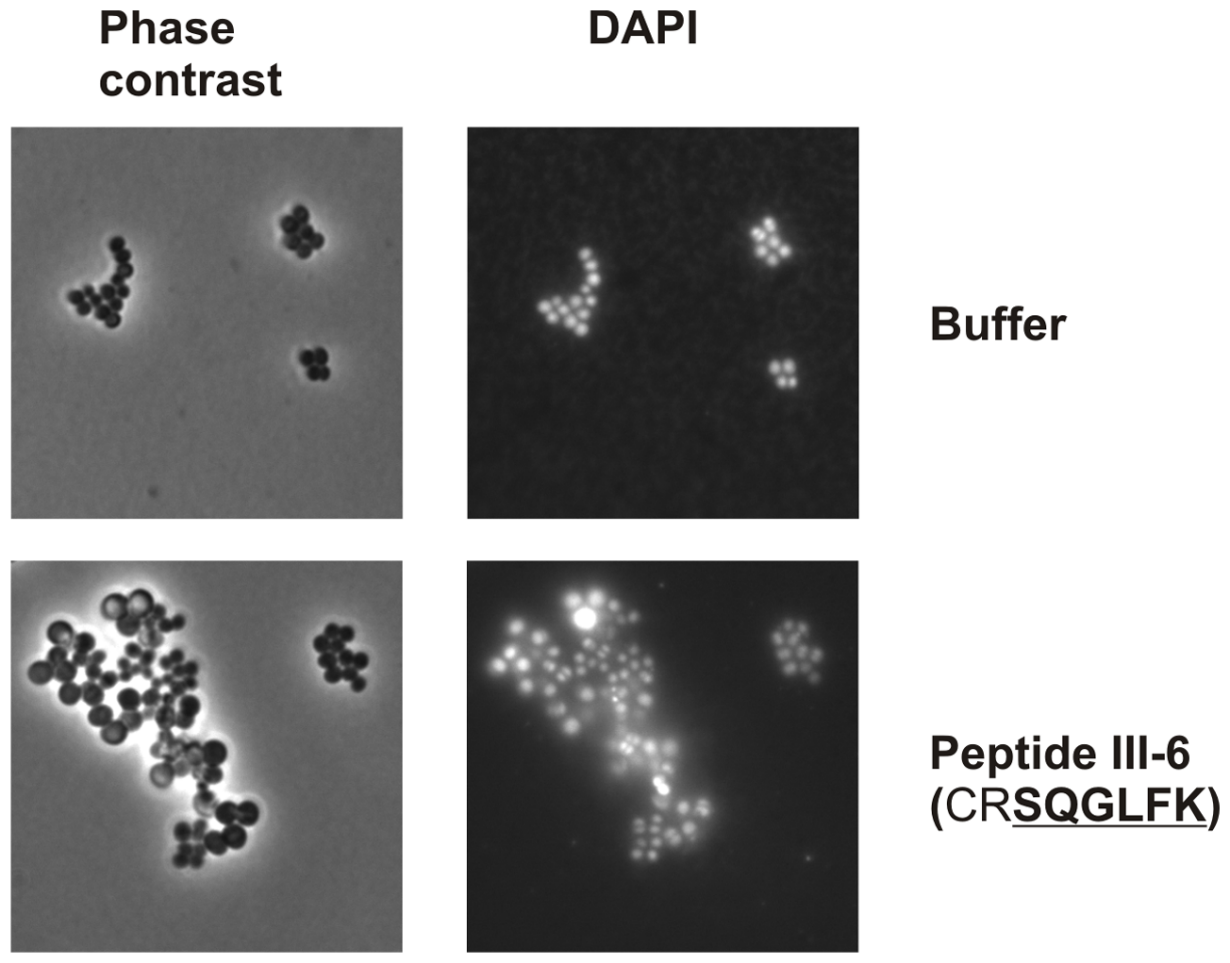
Peptide III-6 affect *S. aureus* cell size, morphology and DNA content. *S. aureus* strain 8325−4 was grown exponentially at 37°C in LB medium. Peptide III-6 was added at 50 μ/m1 (MIC value) and incubation continued for 4 hours. Cells were stained with DAPI prior to fluorescence microscopy (Materials and Methods).

The effect of peptides III-5 and III-6 on cell size and DNA content was visualized by fluorescence microscopy. Five hours incubation with 50 μg/ml of peptide III-6 resulted in increased cell size, with some cells obtaining an almost balloon like appearance (Fig. 7; only data for peptide III-6 is shown). DAPI staining revealed an uneven DNA distribution between cells (Fig. 7). A likely explanation is that peptide III-6, through its inhibitory effect on DNA synthesis, also results in an inability for cells to divide. Similar observations have been made for *E. coli* where failure to complete chromosome replication result in division inhibition and filamentation [38].

### Prolonged exposure to peptides III-5 and III-6 result in cell death

**Figure 8:**
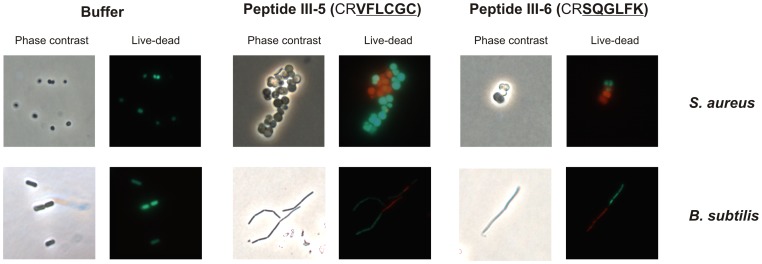
Peptides III-5 and III-6 lead to cell death in *S. aureus* and *B. subtilis*. *S. aureus* strain 8325-4 and *B. subtilis* strain 168 were grown exponentially in LB medium at 37°C. Peptides III-5 and III-6 were added at their MIC values (50 μg/m1) and incubation continued for 6 hours. Cells were live/dead stained with the BacLight system (Materials and Methods) prior to fluorescence microscopy.

To investigate whether peptides III-5 and III-6 acted as bacteriocidal or bacteriostatic agents, we used the *Bac*Light^TM^ bacterial viability kit (Invitrogen Inc). *S. aureus* or *B. subtilis* cultures were grown exponentially in LB and at an optical density OD_600_ = 0.1 peptides were added to a final concentration equal to the MIC value and incubation continued. In the absence of peptide, we observed mostly live (green) cells (Fig. 8). On the other hand, a 6 hour incubation in the presence of peptide III-5 or III-6 resulted in a mixture of dead (red) and live (green) cells (Fig. 8). By quantification of the data obtained we calculated that 28% or 49% of the cells were dead after incubation for 6 hours with peptide III-5 and III-6, respectively. In contrast we only observed 1% dead cells after incubation with a control buffer for 6 hours. Incubation with the cyclic peptides also resulted in enlargement of *S. aureus* cells and filamentation of *B. subtilis*. Thus, long term exposure of *S. aureus* and *B. subtilis* to either peptide results in cell death.

## Discussion

We have used a reverse two hybrid system to identify small cyclic peptides of 8 amino acids that reduce dimerization of the *S. aureus* β-sliding clamp when expressed in *E. coli*. Some peptides were able to enter sensitive bacterial cells and cause arrest of growth and/or cell death due to cessation of bacterial DNA replication.

**Figure 9:**
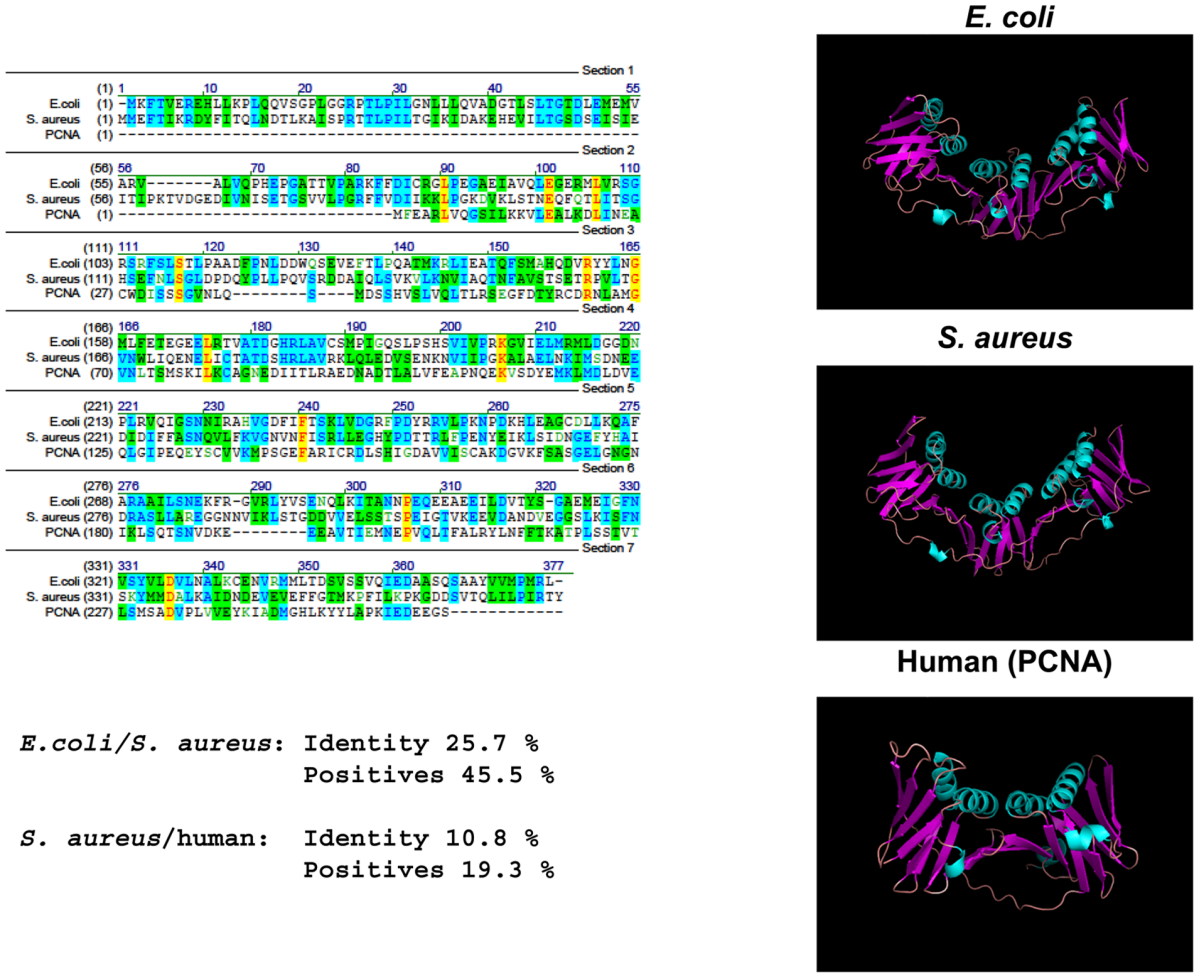
Sequence and structural similarities between β clamps from *E. coli*, *S. aureus* and human. Left: Alignment of *S. aureus* DnaN, *E. coli* DnaN and human PCNA protein sequences using vector NTI deluxe v. 9.0 (Informax Inc.). Yellow: Identical residues in all three species. Blue: Identical residues in two of the three species. Amino acids that are similar in two of the three species are in green. Right: Structure prediction of the same proteins made by the SAM-T08 server (http://compbio.soe.ucsc.edu/papers/sam_doc/sam_doc.html).

The bacterial β-clamp is a homodimer resulting from head to tail association of two three-domain monomers [39],[40] whereas the eukaryotic counterpart, PCNA, is a homotrimer of two-domain monomers [41]. Furthermore the sequence identity between sliding clamps from *S. aureus* and humans is limited to 10.8% (Fig. 9). Altogether this suggests that any compound interfering with the function of the bacterial clamp may not affect the human counterpart, and it has indeed been the target for inhibition in a number of earlier studies. Whereas the previous efforts have focussed on targeting the hydrophobic pocket that interact with other proteins whose action is needed at the fork [14], [17], [42] we have chosen to interfere with dimerization of the clamp. A major concern of ours was that the selection system used was based on a bacterial two-hybrid system [30] and hence carried out in *E. coli*. Any broad spectrum peptide, i.e. targeting both gram positive and gram negative bacteria, would therefore be counterselected due to death of the *E. coli* host. The structure of the *S. aureus* β-sliding clamp is not determined, but when we modelled it with the SAM-T08 server [43] the resemblance to the *E. coli* counterpart was striking (Fig. 9). However the sequence identity was only 25.7% (Fig. 9) and we assumed that our approach could be used to isolate peptides that differentiate between the β-clamp of *S. aureus* and *E. coli*. This turned out to be the case since the peptides isolated were active against the Gram positive bacteria *S. aureus*, *S. epidermidis* and *B. subtilis*, but did not affect growth of the Gram negative *E. coli*. The sequence identity between the β-clamp of *S. aureus* and *S. epidermidis* and *S. aureus* and *B. subtilis* is 93.4% and 54.1% respectively. The isolated peptides were not expected to affect the human β-clamp (PCNA) due to the limited sequence identity to the *S. aureus* counterpart (Fig. 9). This assumption remains to be verified experimentally. None of the identified peptides showed homology to the *S. aureus* β-clamp. This does however not rule out the possibility that they interact with the dimerization interface of DnaN. At present the exact targets on the DnaN protein are not known.

The idea of using peptides as antimicrobial agents is not new. Naturally occurring antimicrobial peptides and their derivatives have for a long time been considered for therapeutic use [44]. Both synthetic linear and cyclic peptides that target intracellular processes have been isolated and extensively characterized [21], [29]. Our approach of using a reverse bacterial two-hybrid system to identify cyclic peptides, generated by the SICLOPPS technology, that interfere with protein-protein interaction was originally developed by Benkovic and co-workers to identify peptides interfering with the function of the ribonucleotide reductase (RNR). RNR is a tetramer consisting of two NrdA and two NrdB subunits and peptides were selected based on their ability to prevent NrdA and NrdB interaction [29]. This is to our knowledge the first attempt to isolate cyclic peptides that target the DNA replication machinery directly. Two lines of evidence suggest that we have been successful in this. First, two peptides with the sequences VFLCGC and SQGLFK (III-5 and III-6; Table 4) inhibited DNA synthesis without affecting protein synthesis when added to a culture of *S. aureus*. Second, overproduction of the DnaN target resulted in resistance towards the same peptides. We therefore consider it unlikely that the antimicrobial effect of these two peptides result from other and unspecific interactions with the bacterial cells.

Peptides III-5, III-6 and III-7 had a somewhat limited activity *in vivo* with MIC values in the range of 20−50 μg/ml. Since the peptides were all efficient in reducing DnaN-DnaN interaction when produced intracellularly (Fig. 3) the MIC values may at least in part reflect difficulties for the peptides in crossing the bacterial membrane. In agreement with this none of these peptides were particularly hydrophobic or cationic (Table 4). At present we do not know how the isolated peptides enter the bacterial cell but given their physical/chemical nature, they are not likely to passively diffuse through the membrane, and a possibility is that they are actively taken up by one of the four oligopeptide permeases present in *S. aureus* cells [45]. This could be tested by construction of deletion mutants lacking one or more of these permeases. We synthesized and tested linear counterparts of the active peptides III-5, III-6 and III-7. None of these linear peptides had any antimicrobial activity (MIC >560 μg/ml) against either *S. aureus* or *S. epidermidis* (not shown; Table 4). This may indicate a decreased proteolytic stability of the linear peptides once inside cells, or may simply reflect a different three-dimentional structure that does not target the β-clamp to the same extent as when circularized. Increased antibacterial activity of peptides due to circularization has previously been described [46].

Addition of peptides III-5 and III-6 to growing and replicating cells resulted in increased expression from the promoter of the SOS regulated *recA* gene. At the replication fork, the β-clamp associated with leading strand synthesis is loaded at initiation of replication and remains associated with the PolIII core enzyme throughout the replication period. However, the appearance of lesions in the DNA may result in replication restart which requires re-loading of the β-clamp [47]. The situation is different for the lagging strand where a new β-clamp is loaded for the synthesis of each Okazaki fragment [48]. Interfering with DnaN dimerization may therefore interfere with both leading and lagging strand synthesis. We suggest that this would initially lead to accumulation of single stranded DNA within the cells which would trigger SOS induction (Fig. 6) and later lead to generation of double stranded breaks. Similarly, chronic SOS induction has been observed in the temperature sensitive *dnaN159* mutant of *E. coli* which is impaired in interaction with PolIII [49]. One of the hallmarks of SOS induction in bacteria is an arrest in cell division resulting from increased expression of the *sfiA*/*sulA* gene [38]. In rod shaped bacteria such as *E. coli* the net result is cell filamentation and this is also what we observed for rod-shaped *B. subtilis* cells after prolonged exposure to DnaN targeting peptides (Fig. 8). For coccoid *S. aureus* and *S. epidermidis* cells we observed that treatment with the same peptides led to enlarged spherical cells and we suggest that this also may result from peptide-mediated arrest in cell division (Fig. 4). We also observed that peptide treated cells varied greatly in DNA content as judged from microscopic studies. These observations are in agreement with uncoupling of leading and lagging strand synthesis which result in failure to complete chromosome replication which may, by segregation failure, explain the appearance of DNA less cells as well as cells containing an increased amount of DNA. A further contribution to the latter could be the occurrence of damage induced DNA replication [50] triggered by strand breaks.

Strand breaks as a result of DnaN inhibition may be sufficient to explain why peptides III-5 and III-6 are bacteriocidal upon prolonged exposure. This situation may be parallel to that elicited by gyrase inhibitors such as ciprofloxacin which trap the gyrase molecule at the DNA cleavage stage and eventually result in formation of double stranded breaks [51].

Clearly the potency of our first generation of peptides targeting the β-clamp of Gram positive bacteria is too poor for direct testing as new antimicrobials. However they may still serve as lead compounds on the way to identify more efficient versions, for example by Quantitative Structure-Activity Relationship (QSAR) modeling, to relate structural characteristics of the peptides to biological activity [52], [53]. A key question is whether their limited activity results from poor entry into bacterial cells, poor interaction with their target or both. It is also our hope that these peptides along with others that target other key interactions between replication proteins will turn out as useful tools for studying DNA replication *in vivo*.

## Materials and Methods

### Bacterial strains, plasmids, primers and growth conditions

All bacterial strains are listed in Table 1. Details on plasmid construction and primer sequences can be found in Table S1 and S2. Cells were grown in LB or TB medium at the temperature indicated. Antibiotics were used at the following concentrations: Ampicillin (100 μg/ml for high copy number plasmids and 50 μg/ml for mini R1 plasmids), Chloramphenicol (20 μg/ml), Kanamycin (50 μg/ml), Erythromycin (10 μg/ml).

*E. coli* strain BTH101Δ*pyrF* was constructed as follows: First, *pyrF* was replaced with the *cat* gene on the chromosome of MG1655 by the procedure described by Datsenko and Wanner [54] using the primers Delta *pyrF* up and Delta *pyrF* down and pKD3 as template. Primer sequences are given in Table S3. Second, the Δ*pyrF*::cat allele was P1 transduced into BTH101. Finally, the chloramphenicol resistance gene were removed as described [54] resulting in BTH101▵*pyrF*.

For construction of *E. coli* strain SC01 (BTH101▵*pyrF, pyrF::lacZ*), *pyrF* was amplified by PCR from MG1655 with primers parAsd *pyrF* up and *pyrF* hindIII down. The sequences of primers are given in Table S3. The PCR product was digested with BamHI and HindIII and inserted into BamHI-HindIII treated pTK532 resulting in pSC533. Plasmid pSC533 contains *pyrF* inserted downstream of the *cat* gene from pKD3 flanked by two FRT sites. Primers *lacZ*-cI up and pSC532 *lacZ* down contains sequences homologous to sites downstream of the *lacZ* promoter on the *E. coli* chromosome. These primers were used to generate PCR fragments containing *pyrF* linked to the *cat* gene and the two FRT sites using pSC533 as template. The PCR product was digested with DpnI and transformed by electroporation into MG1655 and the cells were spread on LB plates containing 20 μg/ml chloramphenicol. The resulting *pyrF*::*lacZ* fusion was transduced into BTH101Δ*pyrF*. The chloramphenicol resistance gene were removed as described [54] resulting in BTH101▵*pyrF*, *pyrF*::*lacZ*.

### Bacterial two-hybrid assay

Bacterial two-hybrid assay was performed as described previously [30]. Derivatives of plasmids pUT18 and p25N encoding replication proteins were constructed by cloning PCR-amplified DNA fragments in frame with the T25 and T18 fragments of *cya*. T18 and T25 fusions were transformed into BTH101, and plated on plates containing 40 μg/ml of 5-bromo-4-chloro-indolyl-β-D-galactoside (X-gal) and relevant antibiotics. Interacting protein fusions resulted in development of blue colonies on the X-gal plates. For β-galactosidase assays, cells were grown exponentially at 30°C in LB supplemented with 0.5 mM IPTG, and β-galactosidase activities were measured as described by [55].

### Western blot analysis

Strain KG22/pSC116 was grown exponentially at 30°C in LB supplemented with 50 μg/ml chloramphenicol. Expression of IntC::DnaA1-86::IntN was induced by addition of 2 mM IPTG. Samples were taken at selected times and fractionated by sodium dodecyl sulfate-polyacrylamide gel electrophoresis (Criterion-Precast Gel; 10 to 20% Tris-HCl; Bio-Rad Inc.). After fractionation the proteins were transferred to a polyvinylidene difluoride membrane, 0.2 μm (Millipore), using a semidry blotting apparatus (Bio-Rad Inc.). The membrane was blocked overnight in TBSa (150 mM NaCl, 50 mM Tris-HCl, pH 10) plus 2% Tween 20, rinsed with TBSa plus 0.05% Tween for 5 min, incubated for 2 h with polyclonal rabbit anti-DnaA antiserum, and washed with TBSa plus 0.05% Tween. The membrane was further incubated for 1.5 h in the presence of porcine anti-rabbit immunoglobulin G antibody conjugated to alkaline phosphatase (DAKO A/S) and washed with TBSa plus 0.05% Tween. The membrane was scanned on a Storm 840 imaging system (Molecular Dynamics Inc.).

### Construction of SICLOPPS libraries

Construction of a 21 amino acid library was done by annealing 100 pmol of each of the three primers Library ClaI-1, Library ClaI-2 and EGFP primer 3 in a 50 μl reaction by heating to 80°C followed by cooling to room temperature over a period of 60 minutes. The sequences of primers are given in Table S3. The annealed oligonucleotides were ligated to 20 μg of pSC118 digested with ClaI and SpeI. The ligation reaction was ethanol precipitated and the library was resuspended in 100 μl TE buffer and transformed into electrocompetent DH10B. This library encodes precursors of cyclic peptides of 21 amino acids of which 6 are randomized. The library contains approximately 900.000 cyclic peptides which are expressed upon addition of IPTG.

### Screening of SICLOPPS library

The 21aa library was transformed into SC01 containing *cya18* and *cya25* fusion plasmids. The amount of 5-FOA was titrated so strains with plasmids encoding interacting partners did not produce any colonies while strains only expressing the Cya18 and Cya25 partners grew. The cyclic peptides were expressed *in vivo* by addition of IPTG to the plates. Plasmid was purified from colonies that grew on 5-FOA plates and re-transformed into SC01 containing the relevant fusion partners.

### Purification of cyclic peptides

Cyclic peptides were purified using the Impact Twin System (New England Biolabs). Overnight cultures of BL21/pSC124G-C and BL21/pSC143 was diluted in TB medium supplemented with 500 μg/ml ampicillin and grown at 30°C. Plasmids pSC124G-C and pSC143 are derivatives of pTWIN1 with the sequence of the cyclic peptide to be purified inserted between the DnaB and Mxe inteins. At an optical density of OD_600_ = 0.8−1.0, IPTG was added to a final concentration of 1 mM. The temperature was decreased to 25°C and induction was carried out for 4 hours. The cyclic peptides were purified as recommended by New England Biolabs with the following exception. The on column cleavage of the Mxe GyrA intein was performed in 25 mM Tris-Hcl, pH 8.5+100 mM NaCl +50 mM MESNA. The cyclic peptide was eluted in 25 mM Tris-Hcl, pH 8.5+100 mM NaCl.

### Synthesis of 8-mers by Fmoc-Solid Phase Peptide Synthesis (SPPS)

2-chlorotrityl chloride Resin-linked amino acids were purchased from Bachem. Fmoc-protected amino acids, NMP, DTT, TFA and DIC were supplied by Iris Biotech. Acetonitrile, acetic acid and DCM were from VWR; HOAt were purchased from GL Biochem Shanghai; DMF was from Milligen; and DIEA, TFE and TIS were supplied by Sigma Aldrich Inc.

The peptides were synthesized manually using 10 ml syringes containing PTFE syringe filters. Differently from nature, the peptides were synthesized from the C- to the N-terminus. The 2-chlorotrityl chloride resin-linked amino acids used for the different peptides and their resin loadings were: H-Cys (Trt) -2-ClTrt-Resin for peptide III-5 (0.57 mmol/g loading); H-Lys(Boc)-2-CLTrt-Resin for peptide III-6 (0.46 mmol/g loading); H-Leu-2-ClTrt-Resin for peptide IV-N8 (0.98 mmol/g loading); H-Asp (OtBu) -2ClTrt-Resin for peptide III-7 (0.83 mmol/g loading). Swelling of resin took place overnight in N-methylmorpholine (NMP), prior to synthesis and NMP was vacuum removed. Fmoc-protected amino acids (4 equivalents) were diluted in 0.4 M 1-hydroxy-7-azabenzotriazole (HOAt) in NMP. Fmoc-deprotection was done by 20% piperidine in *N*-methyl-2-pyrrolidone (NMP) for 3 min, followed by NMP wash (3 times) and another deprotection with 20% piperidine in DMF for 7 min. 1,3-diisopropylcarbodiimide (DIC) (4 equivalents) was added to the Fmoc-protected amino acid in HOAt/NMP solution before each coupling. Coupling and decoupling took place for 2 hours and between these steps wash was performed using NMP. Fmoc-deprotection was done after each recoupling step as described above, followed by NMP wash (10 min).

Linear peptides were obtained by treating the synthesized peptides with 1 ml of TFA:TIS:DTT:H2O (88∶2∶5∶5), accompanied by wash with 4 ml of 95% TFA [56]. They were concentrated under nitrogen evaporation, followed by ether wash (4 ml twice) and lyophilization. The samples were analyzed by analytical RP-HPLC (C_12_ column, 1.5 ml/min flow and linear gradient of A = 0.1% TFA in MilliQ water and B = 0.1%TFA in acetonitrile) and MALDI TOF-MS.

Prior to cyclization, the synthesized peptides were washed twice with ethanol. Resin was removed with 2 ml of AcOH/TFE/ DCM (1∶2∶7) for 2 hours accompanied by wash with the same cocktail mixture (4 ml twice). The crude protected linear peptides were concentrated as described above. They were dissolved in minimal amount of DMF. HBTU (3 equivalents) and DIEA (6 equivalents) in DMF was added stepwise to the dissolved peptides at a time interval of 30 minutes. Cyclization took place overnight and DMF was removed via nitrogen evaporation. Removal of side protecting groups was achieved by treatment with 1 ml TFA:TIS:DTT:H_2_O (88∶2∶5∶5), accompanied by wash with 4 ml of 95% TFA. The crude cyclic peptides were concentrated and precipitated via nitrogen evaporation and ether washing respectively. Preparative RP-HPLC provided with a Vydac C_18_ column was used to purify the crude cyclic peptides, with eluting linear gradient of A = 0.1% TFA in MilliQ water and B = 0.1%TFA in acetonitrile over 85 minutes (flow of 4 ml/min). Fractions were collected and analyzed by analytical RP-HPLC and MALDI TOF-MS.

The matrix used for verification of masses of linear and cyclic peptides via MALDI TOF-MS was α-cyano-p-hydroxycinammic acid, made in water/acetonitrile (7:3) with 0.1%TFA.

### Measurement of DNA synthesis

For measuring DNA and protein synthesis, *S. aureus* strain 8325−4 was grown exponentially at 37°C in LB supplemented with 0.2 ml ^3^H-thymidine (20 Ci/mmol,1 mCi/ml; Perkin Elmer Inc.) and uridine (50 μg/ml). Peptides were added at the MIC at time zero, and samples of 0.5 ml were taken at the indicated timepoints. The cells were lyzed by addition of 1 μl lysostaphin (5 μg/ml) followed by incubation at 37°C for 30 minutes. TCA was added to a final concentration of 10% and the mixtures were incubated on ice for 30 minutes. The TCA insoluble material was transferred to scintillation tubes. The incorporation of ^3^H into DNA was determined using a 1450 MicroBeta TriLux Microplate and Scintillation and Luminiscence counter (Perkin Elmer Inc.).

### Microscopy

*S. aureus* strain 8325-4 or *B. subtilis* strain 168 was grown exponentially at 37°C in LB. Peptides were added to the MIC at time zero and samples were taken for DAPI staining after 4 hours and after 6 hours for the live dead stain (BacLight live/dead staining kit from invitrogen). The cells were stained with the live/dead stain according to the manufacturer. Phase-contrast and fluorescence images were acquired using a Leica DM5000B microscope with a×100 HCX PL APO NA 1.4 objective and a Leica DFC350FX cooled charge-coupled device camera controlled through FW4000 software (version 1.2.1; Leica Microsystems).

## Supporting Information

Figure S1 Peptides III-5 and III-6 does not inhibit protein synthesis.
(DOCX)

Table S1 Plasmids.
(DOCX)

Table S2 Plasmid constructions.
(DOCX)

Table S3 Primers.
(DOCX)
