# Supplementary material for: Cyclic Peptide Inhibitors of the β-Sliding Clamp in Staphylococcus aureus
Source: PLoS One. 2013 Sep 4;8(9):e72273. doi: 10.1371/journal.pone.0072273 (PMC3762901; doi:10.1371/journal.pone.0072273)
Supplement: Figure S1 — Peptides III-5 and III-6 does not inhibit protein synthesis. (DOCX) [file pone.0072273.s001.docx]

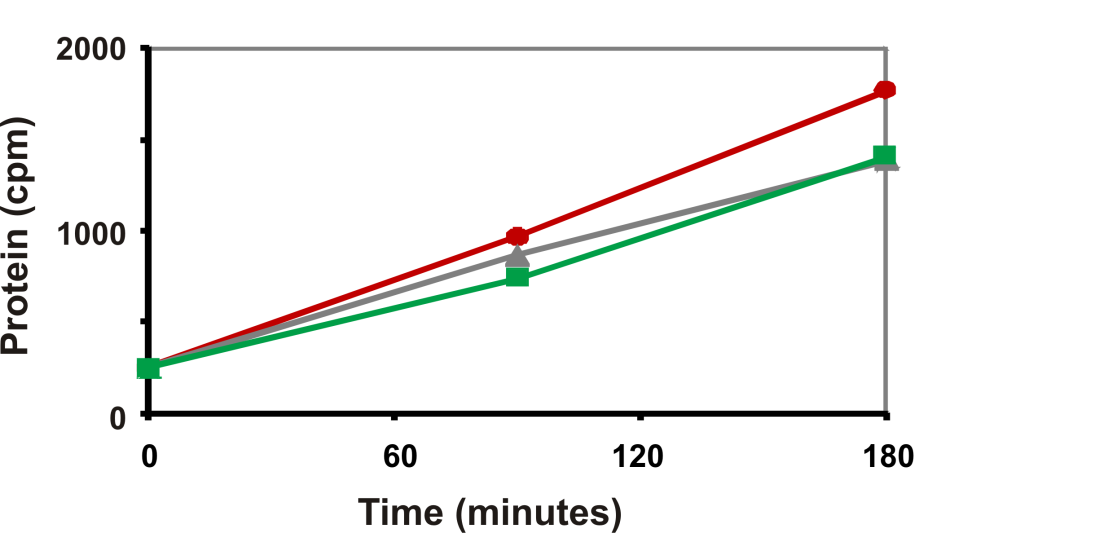


**Figure S1.** Peptides III-5 and III-6 does not inhibit protein synthesis.

*S. aureus* strain 8325-4 was grown exponentially in LB supplemented with ^35^S-methionine. At T=0 peptide III-5 (grey triangles) or III-6 (green squares) was added to a final concentration of 50 μg/ml. Addition of buffer (red filled circles) served as control. Samples were taken at the indicated time-points and incorporation of ^35^S into protein was measured by liquid scintillation counting of TCA precipitated material. The experiment was repeated 3 times with similar result. The result from one experiment is shown.
