# Supplementary material for: Cyclic Peptide Inhibitors of the β-Sliding Clamp in Staphylococcus aureus
Source: PLoS One. 2013 Sep 4;8(9):e72273. doi: 10.1371/journal.pone.0072273 (PMC3762901; doi:10.1371/journal.pone.0072273)
Supplement: Table S1 — Plasmids. (DOCX) [file pone.0072273.s002.docx]

**Table S1. Plasmids used**

All plasmids used in this work are listed. The structure of all plasmids was confirmed by DNA sequencing.

| **Plasmid** | **Properties** | **Reference** |
| --- | --- | --- |
| pKT25 | N-terminal T25 fusion vector | [1] |
| pUT18 | C-terminal T18 fusion vector | [1] |
| pUT18C | N-terminal T18 fusion vector | [1] |
| P25N | C-terminal T25 fusion vector | [2] |
| pKT25-zip | pKT25 containing T25::zip | [1] |
| pUT18C-zip | pUT18C containing T18::zip | [1] |
| pNDM71 | Mini-R1, *bla* | [3] |
| pMGJ25 | pUC, *lacI*, *cat*, pA1/O4/O3 | [4] |
| pNDM220 | Mini-R1 *bla*, *lacI^q^* pA1/O4/O3 | [5] |
| pTWIN1 | pBR, *bla*,T7::CBD::ssp intein::mxe intein::CBD | New England Biolabs |
| pKD3 | *cat* template plasmid | [6] |
| pKD13 | *cat* template plasmid | [6] |
| pKD46 | *bla* λ Red recombinase expression | [6] |
| pCP20 | pSC101*rep^ts^ bla cat* | [6] |
| pTK532 | *cat* template plasmid, pUC, bla | [7] |
| pNW1118 | Plasmid containing the *dnaB* split intein from synechocystis spp | [8] |
| pCN51 | *E. coli, S. aureus* shuttle vector, pCAD | [9] |
| pSC18*dnaA* | pUT18 containing *dnaA*::*cya18* | This work |
| pSC18C*dnaA* | pUT18C containing *cya18*::*dnaA* | This work |
| pSC18*dnaB* | pUT18 containing *dnaB*::*cya18* | This work |
| pSC18C*dnaB* | pUT18C containing *cya18*::*dnaB* | This work |
| pSC18*dnaN* | pUT18 containing *dnaN*::*cya18* | This work |
| pSC18C*dnaN* | pUT18C containing *cya18*::*dnaN* | This work |
| pSC18*dnaX* | pUT18 containing *dnaX*::*cya18* | This work |
| pSC18C*dnaX* | pUT18C containing *cya18*::*dnaX* | This work |
| pSC18*polC* | pUT18 containing *polC*::*cya18* | This work |
| pSC18C*polC* | pUT18C containing *cya18*::*polC* | This work |
| pSC18*holA* | pUT18 containing *holA*::*cya18* | This work |
| pSC18C*holA* | pUT18C containing *cya18*::*holA* | This work |
| pSC18*holB* | pUT18 containing holB::*cya18* | This work |
| pSC18C*holB* | pUT18C containing *cya18*::*holB* | This work |
| pSC25N*dnaA* | P25N containing *dnaA*::*cya25* | This work |
| pSC25K*dnaA* | pKT25 containing *cya25*::*dnaA* | This work |
| pSC25N*dnaB* | P25N containing *dnaB*::*cya25* | This work |
| pSC25K*dnaB* | pKT25 containing *cya25*::*dnaB* | This work |
| pSC25N*dnaN* | P25N containing *dnaN*::*cya25* | This work |
| pSC25K*dnaN* | pKT25 containing *cya25*::*dnaN* | This work |
| pSC25N*dnaX* | P25N containing *dnaX*::*cya25* | This work |
| pSC25K*dnaX* | pKT25 containing *cya25*::*dnaX* | This work |
| pSC25N*polC* | P25N containing *polC*::*cya25* | This work |
| pSC25K*polC* | pKT25 containing *cya25*::*polC* | This work |
| pSC25N*holA* | P25N containing holA::cya25 | This work |
| pSC25K*holA* | pKT25 containing *cya25*::*holA* | This work |
| pSC25N*holB* | P25N containing *holB*::*cya25* | This work |
| pSC25K*holB* | pKT25 containing *cya25*::*holB* | This work |
| pSC7118C | pNDM71 containing *cya18* | This work |
| pSC71*dnaN* | pNDM71 containing *dnaN*::*cya18* | This work |
| pSC533 | pTK532 containing FRT::*cat*::FRT::*pyrF* | This work |
| pSC105 | pNDM220 containing the split intein from pNW1118 | This work |
| pSC111 | Kan resistant version of pNDM220 | This work |
| pSC113 | pSC111 containing the split intein from pNW1118 with one modified restriction site | This work |
| pSC114 | pSC111 containing the split intein from pNW1118 with two modified restriction sites | This work |
| pSC116 | pMGJ25 derivate used for expression of the intC-DnaA1-86-IntN precursor | This work |
| pSC117 | pSC114 encoding intC-DnaA1-86-IntN | This work |
| pSC118 | pMGJ25 containing the split intein from pNW1118. Used for construction of the 21 aa library | This work |
| pSC123 | pTWIN1 derivative, Precursor for purification of linear peptide III-6 | This work |
| pSC124 | pMGJ25 derivative, Precursor for purification of linear peptide III-6 | This work |
| pSC124 G-C | pMGJ25 derivative, Precursor for purification of circular peptide III-6 | This work |
| pSC141 | pCN51 containing pCad::*dnaN* | This work |
| pSC142 | pTWIN1 derivative, Precursor for purification of circular peptide III-5 | This work |
| pSC143 | pMGJ25 derivative, Precursor for purification of circular peptide III-5 | This work |

**References**

1. Karimova G, Pidoux J, Ullmann A, Ladant D (1998) A bacterial two-hybrid system based on a reconstituted signal transduction pathway. Proc Natl Acad Sci U S A 95: 5752-5756.

2. Claessen D, Emmins R, Hamoen LW, Daniel RA, Errington J, et al. (2008) Control of the cell elongation-division cycle by shuttling of PBP1 protein in *Bacillus subtilis*. Mol Microbiol 68: 1029-1046.

3. Christensen SK, Mikkelsen M, Pedersen K, Gerdes K (2001) RelE, a global inhibitor of translation, is activated during nutritional stress. Proc Natl Acad Sci U S A 98: 14328-14333.

4. Overgaard M, Borch J, Jorgensen MG, Gerdes K (2008) Messenger RNA interferase RelE controls *relBE* transcription by conditional cooperativity. Mol Microbiol 69: 841-857.

5. Gotfredsen M, Gerdes K (1998) The *Escherichia coli* *relBE* genes belong to a new toxin-antitoxin gene family. Mol Microbiol 29: 1065-1076.

6. Datsenko KA, Wanner BL (2000) One-step inactivation of chromosomal genes in *Escherichia coli* K-12 using PCR products. Proc Natl Acad Sci U S A 97: 6640-6645.

7. Kruse T, Moller-Jensen J, Løbner-Olesen A, Gerdes K (2003) Dysfunctional MreB inhibits chromosome segregation in *Escherichia coli*. Embo Journal 22: 5283-5292.

8. Williams NK, Prosselkov P, Liepinsh E, Line I, Sharipo A, et al. (2002) *In vivo* protein cyclization promoted by a circularly permuted *Synechocystis* sp. PCC6803 DnaB mini-intein. J Biol Chem 277: 7790-7798.

9. Charpentier E, Anton AI, Barry P, Alfonso B, Fang Y, et al. (2004) Novel cassette-based shuttle vector system for gram-positive bacteria. Appl Environ Microbiol 70: 6076-6085.
