# Supplementary material for: Cyclic Peptide Inhibitors of the β-Sliding Clamp in Staphylococcus aureus
Source: PLoS One. 2013 Sep 4;8(9):e72273. doi: 10.1371/journal.pone.0072273 (PMC3762901; doi:10.1371/journal.pone.0072273)
Supplement: Table S2 — Plasmid constructions. (DOCX) [file pone.0072273.s003.docx]

**Table S2. Plasmid constructions**

| **Plasmid** | **Primers** | **Gene fusion** |
| --- | --- | --- |
| pSC18*dnaA* | pT18C/pT18dnaAup + pT18 dnaA rw | *dnaA::cya18* |
| pSC18C*dnaA* | pT18C/pT18dnaAup + pT25/pT18CdnaArw | *dya18::dnaA* |
| pSC18*dnaB* | pT18C/pT18dnaBup + pT18 dnaB rw | *dnaB::cya18* |
| pSC18C*dnaB* | pT18C/pT18dnaBup + pT25/pT18CdnaBrw | *cya18::dnaB* |
| pSC18*dnaN* | pT18C/pT18dnaNup + pT18 dnaN rw | *dnaN::cya18* |
| pSC18C*dnaN* | pT18C/pT18dnaNup + pT25/pT18CdnaNrw | *cya18::dnaN* |
| pSC18*dnaX* | pT18C/pT18dnaXup + pT18 dnaX rw | *dnaX::cya18* |
| pSC18C*dnaX* | pT18C/pT18dnaXup + pT25/pT18Cdnaxrw | *cya18::dnaX* |
| pSC18*polC* | pT18C/pT18polCup + pT18 polC rw | *polC::cya18* |
| pSC18C*polC* | pT18C/pT18polCup + pT25/pT18CpolCrw | *cya18::polC* |
| pSC18*holA* | SAV1587up + pT18 SAV1587 rw | *holA::cya18* |
| pSC18C*holA* | SAV1587up + SAV1587rw | *cya18::holA* |
| pSC18*holB* | pT18C/pT18holBup + pT18 holB rw | *holB::cya18* |
| pSC18C*holB* | pT18C/pT18holBup + pT25/pT18CholBrw | *cya18::holB* |
| pSC25K*dnaA* | pT25 dnaA up + pT25/pT18CdnaArw | *cya25::dnaA* |
| pSC25K*dnaB* | pT25 dnaB up + pT25/pT18CdnaArw | *cya25::dnaB* |
| pSC25K*dnaN* | pT25 dnaN up + pT25/pT18CdnaArw | *cya25::dnaN* |
| pSC25K*dnaX* | pT25 dnaX up + pT25/pT18CdnaArw | *cya25::dnaX* |
| pSC25K*polC* | pT25 polC up + pT25/pT18CpolCrw | *cya25::polC* |
| pSC25K*holA* | pT25/SAV1587up + SAV1587rw | *cya25::holA* |
| pSC25K*holB* | pT25 holB up + pT25/pT18CholBrw | *cya25::holB* |
| pSC25N*dnaA* | pT25 dnaA up + pT18 dnaA rw | *dnaA::cya25* |
| pSC25N*dnaB* | pT25 dnaB up + pT18 dnaB rw | *dnaB::cya25* |
| pSC25N*dnaN* | pT25 dnaN up + pT18 dnaN rw | *dnaN::cya25* |
| pSC25N*dnaX* | pT25 dnaX up + pT18 dnaX rw | *dnaX::cya25* |
| pSC25N*polC* | pT25 polC up + pT18 polC rw | *polC::cya25* |
| pSC25N*holA* | pT25 holA up + pT18 holA rw | *holA::cya25* |
| pSC25N*holB* | pT25 holB up + pT18 holB rw | *holB::cya25* |

Construction of C-terminal fusions to T18:

*dnaA*, *dnaB*, *dnaN*, *dnaX*, *polC*, *holA* and *holB* were amplified by PCR using the primers shown in Supplementary Table 2. The sequences of primers are given in Supplementary Table 3. Genomic DNA from *S. aureus* strain Mu50 was used as template in the PCR reactions. The PCR products were digested with PstI and BamHI and inserted into pUT18 treated with PstI and BamHI.

Construction of N-terminal fusions to T18:

*dnaA*, *dnaB*, *dnaN*, *dnaX*, *polC*, *holA* and *holB* were amplified by PCR using the primers shown in Supplementary Table 2. The sequences of primers are given in Supplementary Table 3. Genomic DNA from *S. aureus* strain Mu50 was used as template in the PCR reactions. The PCR products were digested with PstI and BamHI and inserted into pUT18C treated with PstI and BamHI.

Construction of N-terminal fusions to T25:

*dnaA*, *dnaB*, *dnaN*, *dnaX*, *polC*, *holA* and *holB* were amplified by PCR using the primers shown in Supplementary Table 2. The sequences of primers are given in Supplementary Table 3. Genomic DNA from *S. aureus* strain Mu50 was used as template in the PCR reactions. The PCR products were digested with PstI and BamHI and inserted into pKT25 treated with PstI and BamHI.

Construction of C-terminal fusions to T25:

*dnaA*, *dnaB*, *dnaN*, *dnaX*, *polC*, *holA* and *holB* were amplified by PCR using the primers shown in Supplementary Table 2. The sequences of primers are given in Supplementary Table 3. Genomic DNA from *S. aureus* strain Mu50 was used as template in the PCR reactions. The PCR products were digested with PstI and BamHI and inserted into p25N treated with PstI and BamHI.

Construction of mini-R1 derivatives of T18 fusions:

The pSC18C and pSC18 plasmids were digested with AatII and PvuII. The fragments encoding the *cya18*::*geneX* and *geneX*::*cya18* fusions were purified and ligated to AatII-Eco721 treated pNDM71.

pSC533: *pyrF* was amplified by PCR from MG1655 with primers parAsd pyrF up and pyrF hindIII down. The PCR product was digested with BamHI and HindIII and inserted into BamHI-HindIII treated pTK532.

pSC105: A PCR product was generated with primers Ssp dnaB KpnI cw and Ssp dnaB speI ccw and pNW1118 as template. A second PCR product was generated with primers Ssp dnaB speI cw and Ssp dnaB ScaI ccw and pNW1118 as template. The sequences of primers are given in Supplementary Table 3. These two PCR products were used as templates in a third PCR reaction with primers Ssp dnaB KpnI cw and Ssp dnaB ScaI ccw. The PCR product were digested with KpnI and ScaI and ligated to pNDM220 treated with EcoRI followed by T4 DNA polymerase and finally KpnI. pSC105 encodes the split intein from pNW1118 with the MluI site changed to a speI site.

pSC111: PCR with primers pOU kan AatII up and pOU kan NdeI down with plasmid pKD13 as template. The PCR product was digested with AatII and NdeI and ligated to pNDM220 treated with AatII and NdeI.

pSC113: PCR with Ssp dnaB KpnI and Ssp dnaB speI ccw with pNW1118 as template. PCR with Ssp dnaB speI cw and Ssp dnaB ScaI ccw with pNW1118 as template. These two PCR products were used as templates in a third PCR reaction with primers Ssp dnaB KpnI cw and Ssp dnaB ScaI ccw. The PCR product were digested with KpnI and ScaI and ligated to pSC111 treated with EcoRI followed by T4 DNA polymerase and finally KpnI. pSC113 encodes the split intein from pNW1118 with the MluI site changed to a speI site. The sequences of primers are given in Supplementary Table 3.

pSC114: PCR with Split intein BamHI cw and pSC113 NheI ccw with pSC113 as template. PCR with pSC113 NheI cw and Split intein XhoI ccw with pSC113 as template. These two PCR products were used as templates in a third PCR reaction with primers Split intein BamHI cw and Split intein XhoI ccw. The PCR product was digested with BamHI and XhoI and ligated to pSC111 treated BamHI and XhoI. pSC114 encodes the split intein from pSC113 but with the EcoRI site changed to NheI. The sequences of primers are given in Supplementary Table 3.

pSC116: pSC117 was digested with BamHI and XhoI. The fragment encoding the split intein in addition to DnaA1-86 was purified and ligated to BamHI-SalI treated pMGJ25

pSC117: PCR with primers dnaA 1-86 NheI up and dnaA 1-86 down. The PCR product were digested with NheI and SpeI and ligated to SpeI-NheI treated pSC114. The sequences of primers are given in Supplementary Table 3.

pSC118: A PCR product was generated with primers Split intein BamHI cw and pSC25 ClaI ccw and pSC105 as template. A second PCR product was generated with primers pSC25 ClaI cw and Split intein XhoI ccw and pSC105 as template. The sequences of primers are given in Supplementary Table 3. These two PCR products were used as templates in a third PCR reaction with primers Split intein BamHI cw and Split intein XhoI ccw. The PCR product was digested with BamHI and XhoI and ligated to pMGJ25 treated BamHI and SalI. pSC118 encodes the split intein from pSC105 but with the EcoRI site changed to ClaI.

pSC123: Primers III-6 cw and III-6 ccw was annealed and ligated to pTWIN1 digested with SapI. The sequences of primers are given in Supplementary Table 3.

pSC124: The inteins of pSC123 is amplified by PCR using primers pTWIN1 BamHI cw and pTWIN HindIII ccw. The sequences of primers are given in Supplementary Table 3. The PCR product was digested with BamHI and HindIII and ligated to pMGJ25 treated BamHI and HindIII.

pSC124 G-C: PCR with pTWIN BamHI cw and III-6 gly-cys R with pSC124 as template. PCR with pTWIN1 HindIII ccw and III-6 gly-cys F with pSC124 as template. These two PCR products were used as templates in a third PCR reaction with primers pTWIN BamHI cw and pTWIN HindIII ccw. The sequences of primers are given in Supplementary Table 3. The PCR product was digested with BamHI and HindIII and ligated to pMGJ25 treated BamHI and HindIII.

pSC141: The *dnaN* gene from *S. aureus* was amplified by PCR with primers pCN51 DnaN F SalI and T25/pT18CdnaN rw using chromosomal DNA from *S. aureus* Mu50 as template. The sequences of primers are given in Supplementary Table 3. The PCR product was digested with SalI and BamHI and inserted into pCN51 opened with SalI and BamHI. pSC141 expresses DnaN from *S. aureus* upon addition of CdCl_2_.

pSC142: Primers pTWIN III-5 cw and pTWIN III-5 ccw is annealed and ligated to pTWIN1 digested with SapI. The sequences of primers are given in Supplementary Table 3.

pSC143: The inteins of pSC142 is amplified by PCR using primers pTWIN1 BamHI cw and pTWIN HindIII ccw. The sequences of primers are given in Supplementary Table 3. The PCR product was digested with BamHI and HindIII and ligated to pMGJ25 treated BamHI and HindIII.
