# Supplementary material for: Cyclic Peptide Inhibitors of the β-Sliding Clamp in Staphylococcus aureus
Source: PLoS One. 2013 Sep 4;8(9):e72273. doi: 10.1371/journal.pone.0072273 (PMC3762901; doi:10.1371/journal.pone.0072273)
Supplement: Table S3 — Primers. (DOCX) [file pone.0072273.s004.docx]

**Table S3. Primer sequences**

| **Primer name** | **Primer sequence (5´- 3`)** |
| --- | --- |
| Delta pyrF up | GCCCATCATCAAGAAGGTCTGGTCATGACGTTAACTGCTTCATCTTCTTCCG TGTAGGCTGGAGCTGCTTCG |
| Delta pyrF down | TCATGCACTCCGCTGTAAAGAGGCGTTGATCGCTTTCAGCGTCTGCGCTGGACATATGAATATCCTCCTTAG |
| lacZ-cI up | CCGGCTCGTATGTTGTGTGGAATTGTGAGCGGATAACAATTTGTGTAGGCTGGAGCTGCTTC |
| pSC532 lacZ down | GACGGCCAGTGAATCCGTAATCATGGTCATAGCTGTTTCCTGCACCTTCATGGTGGTCAGTG |
| parAsd pyrF up | CCCCGGATCCATAAGGAGTTTTATAAATGACGTTAACTGCTTCATCTTC |
| pyrF hindIII down | CCCCAAGCTTTCATGCACTCCGCTGTAAAG |
| pT25 dnaA up | CCCCCTGCAGGGATGTCGGAAAAAGAAATTTGGG |
| pT18C/pT18dnaAup | CCCCCTGCAGGATGTCGGAAAAAGAAATTTGGG |
| pT25/pT18CdnaArw | CCCCGGATCCTTATACATTTCTTATTTCTTTTTCAAG |
| pT18 dnaA rw | CCCCGGATCCTCTACATTTCTTATTTCTTTTTCAAG |
| pT25 dnaN up | CCCCCTGCAGGGATGATGGAATTCACTATTAAAAGAG |
| pT18C/pT18dnaNup | CCCC CTGCAGGATGATGGAATTCACTATTAAAAGAG |
| pT25/pT18CdnaNrw | CCCCGGATCCTTAGTAAGTTCTGATTGGTAAAATTAATTG |
| pT18 dnaN rw | CCCCGGATCCTCGTAAGTTCTGATTGGTAAAATTAATTG |
| pT25 dnaX up | CCCCCTGCAGGGTTGAATTATCAAGCCTTATATCG |
| pT18C/pT18dnaXup | CTGCAGGTTGAATTATCAAGCCTTATATCG |
| pT25/pT18CdnaXrw | GGATCCTCACTCTTCATCTATCACATG |
| pT18 dnaX rw | GGATCCTCCTCTTCATCTATCACATGTAC |
| pT25 holB up | CCCCCTGCAGGGATGGATGAACAGCAACAATTG |
| pT18C/pT18holBup | CCCCCTGCAGGATGGATGAACAGCAACAATTG |
| pT25/pT18CholBrw | CCCCGGATCCCTAACTCACACCCTTAATTAC |
| pT18 holB rw | CCCCGGATCCTCACTCACACCCTTAATTACG |
| pT25 polC up | CCCCCTGCAGGGTTGGCAATGACAGAGCAAC |
| pT18C/pT18polCup | CCCCCTGCAGGTTGGCAATGACAGAGCAAC |
| pT25/pT18CpolCrw | CCCCGGATCCTTACATATCAAATATCGAAAGTTG |
| pT18 polC rw | CCCCGGATCCTCCATATCAAATATCGAAAGTTGAG |
| pT25 dnaB up | CCCCCTGCAGGGATGGGACGACAAGCCTTCG |
| pT18C/pT18dnaBup | CCCCCTGCAGGATGGGACGACAAGCCTTCG |
| pT25/pT18CdnaBrw | CCCCGGATCCTTATCGACTGTCCTCCTCCC |
| pT18 dnaB rw | GGATCCTCTCGACTGTCCTCCTCCC |
| pT25 holA up | CCCCCTGCAGGGATGAGCGACAATATTGTAGC |
| pT18C, pT18 holA up | CCCCCTGCAGGATGAGCGACAATATTGTAGC |
| pT25, pT18C holA rw | CCCCGGATCCTTATAAAGATAGAATGAATAATTCC |
| holA rw | CCCCGGATCCTCTAAAGATAGAATGAATAATTCCAG |
| Split intein BamHI cw | CCCCGGATCCAAAAGGAGGAAAAAACCATGTCTCCGGAAATCGAAAAAC |
| Ssp BsrGI down | CCGCGGAGCTTTTATGTACAATGATGTCGTTG GCCACG |
| pSC25 ClaI ccw | CCTTCTTAAAGCTTTTAATCGATGATAGAGTTGTGAACG |
| pSC25 ClaI cw | CGTTCACAACTCTATCATCGATTAAAAGCT TTAAGAAGG |
| Split intein XhoI ccw | CCCCCTCGAGTTACAGCTGCAGAGAGGAGC |
| Ssp dnaB KpnI cw | CCCCGGTACCTAAAGGGAGGAAAAAACCATGTCTCCGGAAATCGAAAAAC |
| pSC113 NheI cw | GTTCACAACTCTATCGCTAGCTAAAAGCTTTAAGAAG |
| pSC113 NheI ccw | CTTCTTAAAGCTTTTAGCTAGCGATAGAGTTGTGAAC |
| dnaA1-86 NheI up | CCCCGCTAGCGTGTCACTTTCGCTTTGGCAG |
| dnaA1-86 down | CCCCACTAGTCGTTTGCGTCACCGGTTTG |
| pOU kan AatII | CCCCGACGTCGCGCTTTTGAAGCTCACGC |
| pOUkan NdeI down | CCCCCATATGGAATAGGAACTTCAAGATCCCC |
| Ssp dnaB speI ccw | GCAGCCAGACTCACTAGTCGACGTCATATG |
| Ssp dnaB speI cw | CATATGACGTCGACTAGTGAGTCTGGCTGC |
| Ssp dnaB ScaI ccw | CCCCAGTACTTTACAGCTGCAGAGAGGAGC |
| Split intein salI no stop | CCCCGTCGACCAGCTGCAGAGAGGAGCTC |
| Ssp sacII up | TGTACATAAAAGCTCCGCGGATTCTCTGATCAGCCTGGCG |
| Library ClaI-1 | CGATAGTGCAGGTNNBNNBNNBNNBNNBNNBGGAGCTTCTA |
| Library ClaI-2 | ACCTGCACTAT |
| EGFP primer 3 | CTAGTAGAAGCTCC |
| Bib 1 (9 aa) | GTACACAACTCTATCNNBNNBNNBNNBNNBNNBGGCTGCATCTCCGC |
| Bib 2 (9 aa) | GATAGAGTTGT |
| Bib 3 (9 aa) | GGAGATGCAGCC |
| CBD cw SalI | CCCCGTCGACACGACAAATCCTGGTGTATCCG |
| CBD ccw HindIII | CCCCAAGCTTTCATTGAAGCTGCCACAAGGC |
| III-6 cw | AGTCAGGGTCTTTTTAAGGGCTGCATCTCTGGTGATTC |
| III-6 ccw | CTTAAAAAGACCCTGACTGTTGTGAACGATGATGTCGTTG |
| pTWIN1 BamHI cw | CCCCGGATCCAAAAGGAGGAAAAAACCATGAAAATCGAAGAAGGTAAACTG |
| pTWIN1 HindIII ccw | CCCCAAGCTTTCATTGAAGCTGCCACAAGGC |
| III-6 gly-cys F | GACATCATTGTACACAACTGTAGAAGTCAGGGTCTTTTTAAG |
| III-6 gly-cys R | CTTAAAAAGACCCTGACTTCTACAGTTGTGTACAATGATGTC |
| pTWIN III-5 cw | AACTGTAGAGTTTTCTTGTGTGGGTGT |
| pTWIN III-5 ccw | GCAACACCCACACAAGAAAACTCTACA |
| pCN51 DnaN F SalI | CCCCGTCGACGGAGGGTTTATTATGATGGAATTCACTATTAAAAGAG |

(N=A/T/C/G, B= T/C/G)
